# Analysis of Dietary Related Factors of Recurrent Aphthous Stomatitis among College Students

**DOI:** 10.1155/2018/2907812

**Published:** 2018-02-27

**Authors:** Qian Du, Shenglou Ni, Yanling Fu, Sanhai Liu

**Affiliations:** ^1^Beijing University of Chinese Medicine, Beijing 100029, China; ^2^Wenzhou Traditional Chinese Medicine Hospital, Zhejiang 325000, China

## Abstract

**Objective:**

We investigated the occurrence of recurrent aphthous stomatitis (RAS) among college students and its potential influence by dietary habits.

**Methods:**

Study of dietary habits and RAS among students in Beijing University of Chinese Medicine was carried by homemade questionnaire. Multivariate binary logistic regression analysis was used to identify RAS risk factors and explore their relations.

**Results:**

Among 1011 investigated college students, family history (odds ratio (OR) 1.678, 95% confidence intervals (CI) 1.192 to 2.364, *p* < 0.05), bed late (OR 1.515, 95% CI 1.005 to 2.285, *p* < 0.05), frequent thirst (OR 1.842, 95% CI 1.393 to 2.435, *p* < 0.001), and frequent drinking carbonated beverages (OR 1.369, 95% CI 1.029 to 1.821, *p* < 0.05) were independent risk factors for RAS, but preference for nuts (OR 0.607, 95% CI 0.448 to 0.824, *p* < 0.001) was a protective factor. There was no statistical difference in fruit intake between RAS and non-RAS groups (*χ*^2^ = 5.249, *p* > 0.05).

**Conclusions:**

Among college students, frequent drinking carbonated beverages or frequent thirst will increase its possibility, whereas preference for nuts provides protection. In addition, fruit intake does not have a positive effect.

## 1. Introduction

Recurrent aphthous stomatitis (RAS), or recurrent oral ulceration (ROU), is one of the most common oral mucosal diseases. According to worldwide epidemiological data, 2%–66% of the international population is affected [1, 2]. The prevalence of RAS is about 20% in China. The cause of the disease is complicated by its periodicity. In modern medicine, many factors including heredity, oral microbiome disorders, immunologic abnormalities, microcirculatory disturbances, trace element deficiencies, endocrine dyscrasia, and gastrointestinal dysfunction are related to RAS occurrence [3–7], but, to date, the exact etiology and pathogenesis are still unclear. With the change of the whole medical model to preventive medicine, there is an urgency in exploring the diet to interfere with the onset of the disease. Some scholars suggest that the incidence of RAS is closely related to diet, and dietary control has a good application value in its remission by effectively guiding the treatment [8, 9].

In previous studies of RAS relating to diet, some have suggested that spicy food and fried food are risk factors of RAS in the research on the relationship between RAS and bedtime or other pathogenic risk factors [10, 11]. Some scholars choose one or several allergic food(s) in RAS patients by intake or fast test to verify the correlation between RAS and these selected allergic foods [12–14]. Other research focuses on the beneficial effects on RAS by trace elements such as vitamin and dietary fiber and draws a conclusion that more intake of these trace-element-containing food items including grain, dairy product, and fruit can prevent or treat RAS [15]. These studies mentioned above only involve some or some kind of food, and systematic research on daily dietary habits in RAS, especially the possible difference in daily drinks including water intake or beverage, is rarely referred. It is noticed that both milk and grains have been confirmed to cause allergic reactions including RAS [16, 17]. Therefore, it is of great importance to systematically investigate the dietary related factors with RAS.

This study mainly focuses on investigating the prevalence of RAS among college students in Beijing University of Chinese Medicine, analyze the difference of daily dietary habits between RAS patients and the healthy people, and explore its potential influence on RAS. It is beneficial to provide some basis for RAS prevention and treatment in medical students and some guidance in the clinic in future.

## 2. Methods

### 2.1. Subjects

Sixty college students were randomly selected to fill in the self-designed questionnaire after informed consent as a preinvestigation. The prevalence of RAS in preinvestigation was 30%. Based on the formula for estimating sample size, 900 is the baseline. Assume that the nonresponse rate is 10%, 1000 respondents will be suitable. The formula is as follows:

(1) $$\begin{array}{c} n=\frac{U_{1-T/2}^{2}P_{0}\left(1-P_{0}\right)}{d^{2}}, \end{array}$$

where *n* represents the size of sample, *U* is the statistic in confidence level (*T* = 0.05), *P*_0_ is the expected proportion of target population, and *d* is half width of confidence interval (error survey, 0.1-fold of *P*_0_).

### 2.2. Questionnaire

All questions in this questionnaire (see Supplementary Materials (available here)) are based on their own conditions in recent one year. The questionnaire can be divided into three parts, suffering from RAS or not, underlying RAS risk factors, and daily dietary habits. Fifty items are included in this questionnaire. In basic information, age, gender, height, weight, and grade are involved. In Part I, RAS or not, prevalence, therapeutic methods, and remission, and food or flavor worsening or triggering RAS are included. In Part II, wearing dental supplier, family history, allergic history, gastrointestinal disorders, tooth brushing, sleeping, mental status, flavor preference for food, drinking water in warm or cold, dishes mix, and three-meal taking are included. In Part III, habits of taking coffee, tea, alcohol, sweet drink, carbonated beverages, milk, fried food, dessert, ice cream, and fruit are investigated. Exclusion criteria are ① illogical or paradoxical data; ② arbitrary ticking, for instance, ticking the first choice in all items; ③ not answering five or over five items.

### 2.3. Discrimination of RAS

We judge the oral ulceration to be RAS based on its clinical characteristics including periodicity, affected area, and self-limitation. The severity of RAS is graded as the frequency, amount, size, and duration of the ulceration [18].

### 2.4. Cross-Sectional Investigation

The survey was lasted 2 weeks from July 1 to 14, 2017. A total of 1011 college students from Beijing University of Chinese Medicine were enrolled in this study. Stratified sampling and systematic random sampling were successively used. After calculation, there were about 10,000 college students with the ratio of male to female being 9 : 16. The sample proportion was 0.1. The sample size of male and female was 360 and 640, respectively. Four students were classified into a dormitory. The dormitory number was coded at a distance of 10 during sampling. These investigators were all trained about the purpose, significance, and methods of filling in the questionnaire. These questionnaires were collected when the investigators were on the spot in order to deal with doubts in time.

### 2.5. Statistical Analysis

The original data were input into Excel and analyzed by SPSS 20.0. All the data were separately recorded by Qian Du and Sanhai Liu for correction. Based on the acquired literature and outcome in preinvestigation, every risk factor related to RAS was put through binary logistic forward regression analysis to screen out the independent risk factors and control the confounding bias. The regression fitness was judged by Hosmer-Lemeshow test, and the correlation was evaluated by odds ratio (OR) and 95% confidence interval (CI). In Part III, the difference of specific dietary habits in RAS and non-RAS was compared by a chi-square (*χ*^2^) test. The frequency of food or flavor triggering oral ulceration was calculated. Three conditions of fruit intake during ulceration were calculated in their proportions and the difference was analyzed by a chi-square test. *p* < 0.05 was considered as statistically significant.

### 2.6. Ethics

The study protocol was approved by the Ethics Committee of Beijing University of Chinese Medicine (number 2017BZHYLL0308). All participants were informed of the purpose, general contents, and data use.

## 3. Results

### 3.1. Characteristics of the Participants

The number of effective questionnaires was 1011, and the recovery rate was 99.6%. The proportion of male to female was 367 to 644. There were 297 college students with RAS accounting for 29.38%. Among these non-RAS participants, 465 students suffered from oral ulceration but could not be diagnosed as RAS and 249 healthy ones did not have oral ulceration. For RAS students, there was no statistical difference in gender, body mass index (BMI), or grade (Table 1).

### 3.2. Risk Factors Analysis of RAS

All factors including gender, BMI, age, family history, wearing dental braces, gastrointestinal diseases, times of brushing, bedtime at night, duration of brushing time, daily sleep duration, common cold, stress, regular diet, drinking water, and dishes mix were designed as the independent variables. In order to control the confounding bias of dietary factors, dietary contents in Part III were performed with univariate analysis. Besides, frequent intake of sweet drinks, carbonated beverages, and fried food, and preference for nuts which displayed statistical significance was also put into the independent variables for multivariate binary logistic regression analysis. The logistic model was statistically significant (*χ*^2^ = 55.137; *p* < 0.001). Forward (LR) regression indicated that family history, bedtime later than 11 P.M., frequent thirst, frequent consumption of carbonated drinks, and preference for nuts displayed statistical significance. The results showed that the risk of people with family history of RAS was 1.678 times compared with that without RAS family history. People who slept after 11 P.M. suffering from RAS were 1.515 times as high as those sleeping before 11 P.M. For those always felt thirsty, the risk of RAS was 1.842 times in comparison with those who were absent. The risk of RAS was increased by 31.4% compared with those who seldom drank carbonated beverages. People preferring for nuts had a 49.9% lower risk of RAS than those who do not. The goodness of fit was 0.572 in the regression model with a favorable outcome (Table 2).

Table 3 showed that, with regard to daily dietary habits between RAS and non-RAS, there were statistical differences in taking fried food, sweet and carbonated beverage intake, and preference for nuts (all *p* < 0.05). The frequency of food intake or beverage consumption was divided into 5 grades including daily, often, sometimes, seldom, and hardly. Here, “more” refers to the sum of the first three grades, and “less” indicates the last two ones.

### 3.3. Influence of Food and Flavor on RAS

**Figure 1:**
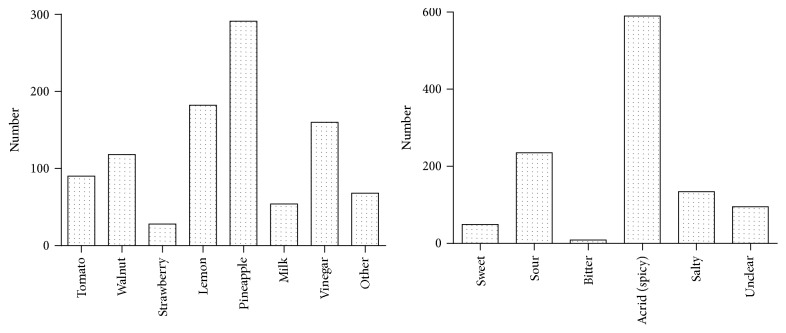
Food or flavor triggering/aggravating RAS.

A previous study suggested that some food might trigger or worsen oral ulceration [19]. Therefore, according to clinical practice and outcome in preinvestigation, 7 daily food and five flavors were selected. The subjects were composed of 297 RAS and 465 students who had oral ulceration in recent one year. Based on their own conditions, after analysis, the top three foods triggering or worsening oral ulceration were pineapple (291/762), lemon (182/762), and vinegar (160/762). The food flavor was displayed from high to low as acrid (spicy) > sour > salty > sweet > bitter accordingly (Figure 1).

Furthermore, we compared the selection differences of pineapple and spicy (flavor) between RAS group and non-RAS group. There was no significant difference in pineapple or spicy (flavor) selection between the two groups (both *p* > 0.05). The result displayed that pineapple and spicy (flavor) were considered to trigger or worsen oral ulceration in the two groups (Table 4).

In addition, we found an interesting phenomenon during onset of oral ulceration-some ate more or less fruits on purpose, and others did not care about this. Among these 762 college students with oral ulceration, the number of eating more and less fruits was 401 (52.6%) and 47 (6.2%), respectively. The rest 314 students did not care about the influence of eating fruits on oral ulceration (Table 5).

After a chi-square test, no statistical difference was displayed (*χ*^2^ = 5.249; *p* = 0.072), which indicated no great difference in fruit intake by the three ways during the onset of oral ulceration between RAS group and non-RAS group. It is not conclusive that fruit intake has an active effect on oral ulceration healing. With reference to the result of frequency of daily fruit intake in Table 3, the conclusion that fruit intake decreases the prevalence of RAS cannot be drawn.

## 4. Discussion

Recurrent aphthous stomatitis is categorized into oral ulcer* (kou chuang)* or oral erosion* (kou mi)* in traditional Chinese Medicine, which was recorded in* Internal Canon of Huangdi*. Oral diseases are directly associated with dietary factors. We endeavor to explore the correlation between RAS and dietary factors.

### 4.1. Prevalence of RAS

Our study showed that the prevalence of RAS in college students from Beijing University of Chinese Medicine (North China) was 29.38%. Zhong et al. report the RAS prevalence in college students in Xinjiang Medical colleges (Northwest, China) was 13.1% in 2014 [20]. Shi et al. report the RAS prevalence in college students in Wuhu City (East China) in 2015 was 23.30% [11]. In Sichuan University (Southwest, China), RAS prevalence in 2014 reported by Ma et al. was 53.2% [10]. The RAS prevalence was 59.43% in college students in Guangzhou Medical College (South China) by Xie et al. in 2009 [21]. The above research indicated that RAS prevalence in college students in Beijing University of Chinese Medicine was high, and the oral health status was not satisfactory. RAS prevalence was varied in different regions in China as a sequence of South China, Southwest, North China, East China, and Northwest. It was assumed that difference RAS prevalence might be associated with geographical and environmental factors and dietary factors. But in Northwest regions, more occurrences of dry mouth and thirst but with a lower RAS prevalence could be explained as drinking more water, which still needs further confirmation. Some research has proved bad habit of tooth brushing and stress were risk factors of RAS [22, 23], but no great difference between RAS group and non-RAS group, which suggested that oral disease prevention and health education in college student should be intensified.

### 4.2. Correlation between RAS and Dietary Factors

Our finding displayed that frequent consumption of carbonated beverages was an independent risk factor for RAS. The prevalence of RAS was higher in those who frequent drinking of sweet drinks, carbonated beverages, and higher intake of fried foods. The habits of sweet and acidic intake can lead to changes in pH in the mouth. Normally, the saliva is neutral and can maintain the stability of the oral chemical environment, while sweet or carbonated beverages lower the pH and change the oral environment. It is reported that stomatitis is more likely to occur when pH in the mouth is abnormal [24]. Teeth invasion by soft drink leads to excessive detrition of dental enamel. Carbonated drink softens the enamel surface leading to extremely rough, porous, and alveolate demineralization, which then causes the wear of soft tissues in the mouth [25–27]. At the same time, it is displayed that preference for nuts is a protective factor for RAS, which could be explained by nuts being rich in vitamins A, B, and E and proteins. If in shortage, they will trigger RAS [28]. Additionally, antioxidant components including flavone and phenolic acids (tannin) in nuts can clear free radicals and have effects of antisepsis and anti-inflammation. Unsaturated fatty acids in nuts may have some lubrication effect on the oral mucosa and reduce the incidence of RAS [29]. Fried and spicy foods are in high calories resulting in a temporary shortage of free moisture in the mouth, which disturbs the balance between free water and bound water in the surface of oral mucosa, intensifying the energy metabolism by heat increase, and symptoms of uprising of fire including aphtha ensue [30]. Reduction of saliva in the mouth fails in protection of oral mucosa. Spicy food has a stimulating effect on the oral mucosa. According to the data in Figure 1, pineapple may induce or aggravate oral ulcers, which may be related to glycosides in the pineapple stimulating oral mucosa and protease leading to allergic reactions in some people [31, 32].

### 4.3. Correlation between RAS and Dry Mouth

This study revealed that people who often present with dry mouth and thirst are more prone to develop RAS. Through clinical trials, Gu et al. found that the secretion by salivary glands in RAS patients is inferior to that in the healthy, and RAS is in spontaneous recovery when secretion becomes normal [33]. It is indicated that the protective characteristics of saliva are not limited to the traditional concepts of cleaning, washing, lubrication, buffering, and supplementing minerals, but also in the transportation of a variety of antiviral, antibacterial, and antifungal factors, and polypeptide growth factors. From this, great attention should be paid to research on salivary secretion in the clinical treatment of RAS.

Usually, people believe oral ulceration to be due to uprising of fire and thirst and preference for cold drinks which are common symptoms. But in our investigation, among RAS students, some students were absent of thirst. Some were thirsty and preferred drinks. Others felt thirsty but were reluctant to have a drink. For those who were thirsty and preferred drinks, the drinks could be divided into hot and cold, which indicates cold and heat patterns, respectively, from the perspective of eight-principle pattern identification. This is helpful for RAS prevention and treatment by traditional Chinese medicine.

### 4.4. Correlation of RAS and Fruits

Our study revealed that, in both RAS and non-RAS groups, daily consumption of fruits was high (Table 3). In traditional Chinese medicine, it is deemed that fruits have cold or heat properties. People with cold deficiency constitution will worsen their conditions by eating cold fruits, while, for those with a heat constitution, taking fruits in heat property will aggravate the symptoms including oral ulcers. Based on Table 5, 52.6% participants will take more fruits on purpose during oral ulceration, which may be related to the idea of rich vitamins in fruits being effective on ulcer healing or intake of more fruits being able to subdue fire and supplement water for oral ulcer being caused by fire [34], but these views are not entirely correct. Lalla et al. confirmed that daily intake of multivitamin as a supplementation could reduce the frequency or duration of RAS attack by a randomized, double-blind trial. It is suggested that multivitamin as a supplement should not be used on RAS prevention by clinical physicians [35]. This can be verified by pineapple triggering or aggravating oral ulcer in our study. The etiology of oral ulcer is related to not only fire, but cold as well. Therefore, in the treatment of RAS, sole focus on selecting herbs with effects of clearing heat, dispersing fire, and resolving toxins is not entirely appropriate, as formulas in warming and supplementing is also useful in clinic [36, 37]. Meanwhile, fruit intake during oral ulcer should be done with caution.

## 5. Conclusions

Our findings indicate that family history, bedtime later than 11 P.M., dry mouth and thirst all the time, and frequent intake of carbonated beverages were independent risk factors of RAS. It is suggested that college students, especially those suffering from RAS, should pay attention to the family history and seek treatment. It is advised not to stay up late. In the aspect of dietary habits, frequent water drinking, reduction of intake on carbonated drinks, and slight increase of nuts intake are advocated. At the same time, fried food and sweet drinks should be reduced. Large quantities of fruits intake do not play a positive role in the prevention and treatment of RAS, so one should be cautious on ingestion and selection of fruits. Pineapple and spicy foods may stimulate or aggravate oral ulcers, people with RAS or during oral ulceration are best to avoid these foods.

## Figures and Tables

**Table 1:** General characteristics of 1011 college students.

Items	RAS		*χ* ^2^	*p*
	Yes	No		
Gender				
Male	116 (31.61%)	251 (68.39%)	1.3819	0.2398
Female	181 (28.11%)	463 (71.89%)		
EB				
Bachelor	179 (30.86%)	401 (69.14%)	2.1880	0.3349
Master	102 (26.70%)	280 (73.30%)		
Doctorate	16 (32.65%)	33 (67.35%)		
BMI^*∗*^				
Underweight	56 (31.82%)	120 (68.18%)	1.0236	0.7956
Normal	217 (28.78%)	537 (71.22%)		
Overweight	20 (31.25%)	44 (68.75%)		
Obesity	4 (23.53%)	13 (76.47%)		

*Note.* RAS: recurrent aphthous stomatitis. EB: education background. BMI: body mass index. ^*∗*^BMI < 18.5 is considered as underweight; 18.5 ≤ BMI < 25 as normal; 25 ≤ BMI < 28 as overweight; 28 ≤ BMI < 32 as obesity.

**Table 2:** Multivariate binary logistic regression of RAS^*∗*^.

Risk factors	*B*	S.E.	Wald	*p*	OR	95% CI
Family history	0.518	0.175	8.784	0.003	1.678	1.192 to 2.364
Bed late	0.416	0.209	3.937	0.047	1.515	1.005 to 2.285
Frequent thirst	0.611	0.143	18.368	0.000	1.842	1.393 to 2.435
FDCB	0.314	0.146	4.646	0.031	1.369	1.029 to 1.821
Preference for nuts	−0.499	0.155	10.301	0.001	0.607	0.448 to 0.824

*Note.* FDCB: frequent drinking carbonated beverages. ^*∗*^LR regression model.

**Table 3:** Frequency or hobby of food intake.

Items	RAS		*χ* ^2^	*p*
	Yes	No		
Coffee			0.2644	0.6071
More	63	162		
Less	234	552		
Tea			1.2960	0.2550
More	122	266		
Less	175	448		
Alcohol			2.2208	0.1362
More	47	88		
Less	250	626		
Sweet drink			6.1452	0.0132
More	178	367		
Less	119	347		
Carbonated beverage			6.7377	0.0094
More	147	290		
Less	150	424		
Dairy product			1.0642	0.3023
More	201	459		
Less	96	255		
Fried food			4.4995	0.0339
More	176	371		
Less	121	343		
Desserts			2.3499	0.1253
More	199	442		
Less	98	272		
Ice cream			0.0440	0.8338
More	134	317		
Less	163	397		
Fruit			0.1241	0.7247
More	238	579		
Less	59	135		
Nuts			12.7704	0.0017
like	82	271		
Equivocal	158	351		
Dislike	57	92		
Raw and cold food			2.3452	0.3096
Like	54	138		
Equivocal	141	302		
Dislike	102	274		

**Table 4:** Pineapple and spicy food triggering/aggravating RAS or not.

Items	RAS		*χ* ^2^	*p*
	Yes	No		
Pineapple			0.9639	0.3262
Yes	107	184		
No	190	281		
Spicy food			0.1313	0.7171
Yes	232	358		
No	65	107		

**Table 5:** Comparison of fruit intake during oral ulceration between RAS group and non-RAS group.

Groups	I^*∗*^	II	III	Sum
RAS group	159 (53.5%)	25 (8.4%)	113 (38.1%)	297
Non-RAS group	242 (52.1%)	22 (4.7%)	201 (43.2%)	465
Sum	401 (52.6%)	47 (6.2%)	314 (41.2%)	762

*Note*.^*∗*^I refers to eating more fruits on purpose; II refers to eating less fruits on purpose; III refers to intension-free fruit consumption.

## Additional Points

*Limitation.* We only focused on college students from bachelor to doctor degrees in one university in Beijing without considering the age factor. The outcomes were obtained based on the data collected in July 2017 (in summer). No other relative documents referring to the influence of different seasons on RAS were reported. Therefore, one should be cautious when applying our outcomes to explain RAS during other seasons. In addition, large-scale social survey besides college students is necessary for comprehensive exploration of RAS's current status and corresponding therapeutic measures.

## Supplementary Materials

Supplementary Materials Fifty items including general demographic information, dietary habits, life style, and so forth on RAS were investigated in this questionnaire.
