# Supplementary material for: Analysis of Dietary Related Factors of Recurrent Aphthous Stomatitis among College Students
Source: Evid Based Complement Alternat Med. 2018 Feb 27;2018:2907812. doi: 10.1155/2018/2907812 (PMC5848137; doi:10.1155/2018/2907812)
Supplement: Supplementary Materials — Fifty items including general demographic information, dietary habits, life style, and so forth on RAS were investigated in this questionnaire. [file 2907812.f1.pdf]

# Questionnaire on Correlation between Recurrent Aphthous Stomatitis and Dietary Factors

**Serial No.: __________**

**Date of Survey: _________**

**Investigator: _________**

Dear schoolmates,

We are grateful for your help in filling this questionnaire. In this study, the correlation between recurrent aphthous stomatitis and dietary factors will be explored. All information provided will be held in strict confidence and will not be used for purposes outside the scope of our research. Thank you for your support and cooperation. We wish you good health!

## General Information

| | |
|---|---|
| Gender: Male/Female | Year of Birth: 19____ |
| Height: ____ m | Weight: ___ kg |
| Education Background: Bachelors, Masters, Doctorate | |

## Part I: Relative Information of Oral Ulcers (based on the past year)

☆ Have you suffered from oral ulcers?

A. Yes B. No

[If yes, please answer all the following questions; if no, please start from Part II.]

A01. How often do you suffer from oral ulcers?

A. every 2 weeks B. every month C. every 2 months

D. every 3 months E. every 6–12 months

A02. How many ulcers are there during each episode?

A. 1–2 B. 3–5 C. ≥ 6

A03.What is the size of ulcers?

A. Like needle point (diameter ≤ 1 mm)

B. Like mung bean (diameter ≤ 5 mm)

C. Like soybean (diameter ≤ 10 mm)

D. Others: ________

A04. How long does each episode last?

A. < 7 days B. 7–14 days

C. 14–30 days D. ≥ 30 days

A05. How do you treat your ulcers? (multiple choices are allowed)

A. Only traditional Chinese herbal medicine

B. Only western medicine

C. Combination of traditional Chinese and western medicine

D. Acu-moxibustion and cupping

E. Buccal or chewable tablets

F. No treatment

A06. What do you think of the method(s) selected in item A05?

A. Invalid B. Fair C. Marked effect

A07. Do you increase fruit consumption during an episode of ulcers?

A. Yes B. No

A08. Do you inhibit fruit consumption during an episode of ulcers?

A. Yes B. No

A09. In your own situation, which food(s) would trigger or aggravate the ulcers? (multiple choices are allowed)

A. Tomato B. Walnut C. Strawberry D. Lemon
E. Pineapple F. Milk G. Vinegar H. Other_______

A10. Which flavor(s) would aggravate your ulcer? (multiple choices are allowed)

A. Sweet B. Sour C. Bitter D. Acrid (spicy) E. Salty F. Unclear

A11. For oral ulceration, which method(s) do you adopt to alleviate pain? (multiple choices are allowed)

A. Drink water frequently B. More sleeping
C. More intake of vegetable D. More intake of fruit (i.e._____)
E. Other________________

A12. What do you think of the remission in item A11?

A. Invalid B. Fair C. Marked effect

## Part Ⅱ: General Information (Based on Your Situations in Recent One Year)

B01. Do you wear tooth socket or other dental braces?

A. Yes B. No

B02. Have you been diagnosed with gastrointestinal diseases such as gastritis, gastric ulcer, and enteritis, etc. in a medical institution?

A. Yes B. No

B03. Do you have a family history of recurrent aphthous stomatitis?

A. Yes B. No

B04. How often do you brush your teeth each day?

A. 0–1 time B. 2–3 times C. other______

B05. Hong long do you brush your teeth for?

A. Half a minute B. 1 minute C. 2 minutes D. 3 minutes E. other____

B06. On average, how many hours do you sleep?

A. < 6 hours B. 6–7 hours C. 7–8 hours D. 8–9 hours E. > 9 hours

B07. What time do you sleep?

A. Before 11 P.M. B. 11 P.M.–12 P.M. C. 12 P.M.–1 A.M.
D. 1 A.M.–2 A.M. E. After 2 A.M.

B08. How often do you have a common cold in the past year?

A. 0–1 time B. 2–3 times C. 4–5 times D. > 5 times

B09. In your study or daily life, how often do you feel stressed, nervous, or anxious?

A. Never B. Seldom C. Sometimes D. Often E. Daily

## Part III: Dietary Habits (Based on Your Situations in Recent Half a Year)

C01. Please rank the following flavors in the order of 1 to 6 based on your taste preference? ("1" refers to like best, and "6" refers to like least)

( ) Acrid (spicy)
( ) Salty
( ) Sour
( ) Sweet
( ) Bitter
( ) Bland

C02. Do you always feel thirsty or have a dry mouth?

A. Yes B. No

C03. Do you like drinking water?

A. Like B. Equivocal C. Dislike

C04. What is your water consumption preference?

A. Like cold water B. Like hot water
C. Like warm water D. No preference.

C05. How many times do you have breakfast each week?

A. 0–1 time B. 2–3 times C. 4–5 times D. 6–7 times

C06. How many times do you have supper each week?

A. 0–1 time B. 2–3 times C. 4–5 times D. 6–7 times

C07. When do you normally have your supper?
A. 5 P.M.–7 P.M. B. 7 P.M.–8 P.M. C. After 8 P.M. D. No supper

C08. What is your ratio of meat to vegetable intake?
A. Only vegetable or a modicum of meat
B. The ratio of meat and vegetable is 3:7
C. Half and half D. Mainly meat

C09. What kind(s) of meat do you consume in your daily life? (multiple choices are allowed)
A. No meat at all B. Freshwater fish C. Beef D. Mutton
E. Pork F. Chicken G. Duck H. Seafood

C10. Do you like consuming raw food, such as salad vegetable, sashimi, etc.
A. Like B. Equivocal C. Dislike

C11. Do you like consuming walnut, peanut, melon seeds, cashew nut, and pine nut, etc.?
A. Like B. Equivocal C. Dislike

C12. How often do you consume coffee?
A. Daily B. Often C. Sometimes D. Seldom E. Hardly

C13. How often do you consume tea?
A. Daily B. Often C. Sometimes D. Seldom E. Hardly

C14. How often do you consume alcohol?
A. Daily B. Often C. Sometimes D. Seldom E. Hardly

C15. How often do you consume sweet drinks such as milk tea and juice?
A. Daily B. Often C. Sometimes D. Seldom E. Hardly

C16. How often do you consume carbonated beverages such as Cola, Sprite, and $^{+}$C, etc.?
A. Daily B. Often C. Sometimes D. Seldom E. Hardly

C17. How often do you consume dairy products such as milk and goat milk, etc.?
A. Daily B. Often C. Sometimes D. Seldom E. Hardly

C18. How often do you consume fried or broiled food?
A. Daily B. Often C. Sometimes D. Seldom E. Hardly

C19. How often do you consume desserts?

A. Daily B. Often C. Sometimes D. Seldom E. Hardly

C20. How often do you consume cold foods such as ice cream, iced beverage, and frozen yogurt, etc.?

A. Daily B. Often C. Sometimes D. Seldom E. Hardly

C21. What is your usual fruit intake?

A. Daily B. Often C. Sometimes D. Seldom E. Hardly

C22. What is your fruit consumption preference?

A. Eat room temperature fruit

B. Eat after warming the fruit

C. Eat straight out from cold storage

C23. Among the following, select your favorite category of fruits.

A. Fruits with cold or cool property, like pear, watermelon, banana, kiwi fruit, and grapefruit, etc.

B. Fruits with neutral property, like grape, plum, and jujube, etc.

C. Fruits with warm or hot property, like orange, apricot, peach, cherry, lychee, pawpaw, and loquat.

***Thank you for your support and cooperation!***
